# Increased Intra-Abdominal Pressure During Laparoscopic Pneumoperitoneum Enhances Albuminuria via Renal Venous Congestion, Illustrating Pathophysiological Aspects of High Output Preeclampsia

**DOI:** 10.3390/jcm9020487

**Published:** 2020-02-11

**Authors:** Pauline Dreesen, Melanie K. Schoutteten, Nele Vande Velde, Iris Kaminski, Line Heylen, Bart De Moor, Manu L.N.G. Malbrain, Wilfried Gyselaers

**Affiliations:** 1 UHasselt—Hasselt University, Faculty of Medicine and Life Sciences, Department of Physiology, Limburg Clinical Research Center, 3590 Diepenbeek, Belgium; 2 Future Health, Ziekenhuis Oost-Limburg, 3600 Genk, Belgium; 3 Department of Nephrology, Catharina Ziekenhuis, 5623 EJ Eindhoven, The Netherlands; 4 Department of Nephrology, Ziekenhuis Oost-Limburg, 3600 Genk, Belgium; 5 Department of Nephrology, Jessa Ziekenhuis, 3500 Hasselt, Belgium; 6 Department of Intensive Care, Universitair Ziekenhuis Brussel, 1090 Jette, Belgium; 7 Faculty of Medicine and Pharmacy, Vrije Universiteit Brussel, 1050 Brussels, Belgium; 8 Department of Obstetrics & Gynecology, Ziekenhuis Oost-Limburg, 3600 Genk, Belgium

**Keywords:** Intra-abdominal pressure, preeclampsia, proteinuria, venous congestion

## Abstract

Intra-abdominal hypertension (IAH) causes severe organ dysfunction. Our aim is to evaluate the effect of increased intra-abdominal pressure (IAP) on renal function, hypothesizing that venous congestion may increase proteinuria and fluid retention without endothelial dysfunction. Three urine samples were collected from 32 non-pregnant women undergoing laparoscopic-assisted vaginal hysterectomy (LAVH) and from 10 controls placed in Trendelenburg position for 60 min. Urine sampling was done before (PRE), during or immediately after (PER), and two hours after (POST) the procedure. Urinary albumin, protein and creatinine concentrations were measured in each sample, and ratios were calculated and compared within and between groups. During LAVH, the albumin/creatinine ratio (ACR) increased and persisted POST-procedure, which was not observed in controls. A positive correlation existed between the LAVH duration and the relative change in both ACR and protein/creatinine ratio (PCR) PER- and POST-procedure. Iatrogenic IAH increases urinary ACR and PCR in non-pregnant women via a process of venous congestion. This mechanism might explain the presentation of one specific subtype of late-onset preeclampsia, where no drop of maternal cardiac output is observed.

## 1. Introduction

Intra-abdominal pressure (IAP) arises due to interactions between contents within the abdominal cavity and mechanical properties of the abdominal wall [1,2], and strongly correlates to intravesical pressure [3]. It is most often estimated as the pressure in a urinary bladder catheter after the evacuation of all urine and instillation of 25cc saline at end-expiration [4,5]. A rise in IAP lowers the abdominal perfusion pressure (i.e., defined as the difference between the mean arterial pressure and IAP) and subsequently blood supply of organs and vessels in or near the abdomen [6]. Once IAP reaches values equal to or greater than 12 mmHg, which is consistent with intra-abdominal hypertension (IAH), the abdominal venous system is compressed, leading to a systemic decrease in venous return and a reduced cardiac preload and output. Largest reductions in blood flow have been reported at the level of the renal and mesenteric arteries [1]. Reduced renal arterial perfusion causes a lower glomerular filtration rate and activation of the juxtaglomerular apparatus and the renin-angiotensin system. This results in oliguria, albuminuria, hypervolemia and hypertension [7,8,9,10]. In the retrograde direction, the reduced venous blood flow further decreases the capillary blood flow and enhances ischemia. The associated tubular damage can effectuate the proteinuria and effective renal plasma flow [6,8,11]. If the abdominal wall is at maximum distension, compensated IAH may convert to uncompensated abdominal compartment syndrome with disseminated organ failure [12].

The Hasselt University Research Team on Maternal Venous Hemodynamics explores the changes of maternal intra-abdominal venous flow during normal and uncomplicated pregnancies. They observed that the Valsalva maneuver induced increase of IAP causes hepatic venous Doppler flow changes similar to those observed in uncomplicated third trimester pregnancy [13]. Next to this, it was also observed that venous hemodynamic dysfunction is an intrinsic part of the pathophysiology of preeclampsia (PE), but not gestational hypertension [14,15]. Today, different types of PE are considered, one of which presents near term without reduction of cardiac output [16]. We wondered whether the pathophysiology of this high output type of PE could be explained via increased IAP and subsequent venous congestion.

The aim of the present study is to investigate the role of increased IAP on proteinuria and albuminuria. We used a model of pneumoperitoneum-induced increase of IAP in non-pregnant women undergoing elective laparoscopic procedures to evaluate proteinuria and albuminuria before, during and after pneumoperitoneum, in comparison to a control group.

## 2. Materials and Methods

This prospective study was approved by the institutional review board of the local ethical committee of Ziekenhuis Oost-Limburg (ZOL, Genk, Belgium—Reference: 16/081U). The study was conducted in accordance with good clinical practice and the Declaration of Helsinki. Oral and written informed consent was obtained from all subjects before inclusion. All subjects were included at ZOL.

### 2.1. Study Population

The study population consisted of an intervention group and a control group. Non-pregnant women (age ≥18 years) undergoing laparoscopic-assisted vaginal hysterectomy (LAVH) were included in the intervention group (i.e., defined as the LAVH group). Women were excluded if they received treatment for oncologic and/or renal diseases. To control for positioning in Trendelenburg (i.e., 15° head-down position), a group of healthy female volunteers were placed in 15° head-down position for 60 min (i.e., defined as the Trendelenburg group). Baseline demographic and clinical characteristics were collected for all subjects: Age, weight, height, body-mass index (BMI), smoking status, pre-existing co-morbidities, parity and medication use.

### 2.2. Study Procedure

For all laparoscopic interventions, an indwelling urinary catheter was placed in situ at least two hours before transport to the operating theatre. All LAVH procedures were performed under general anesthesia and in Trendelenburg position. Standard laparoscopic pneumoperitoneum was applied with device controlled insufflation of CO_2_ gas to IAP pressures between 11 and 14.7 mmHg. There was no anesthesia, peritoneal CO_2_ insufflation or a urinary catheter involved in the Trendelenburg group.

### 2.3. Urine Sampling

**Figure 1:**
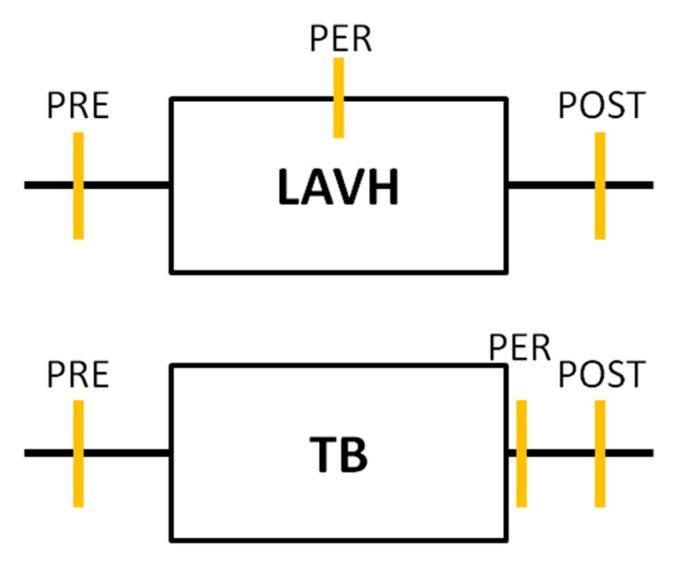
Timeline of the urine analyses performed in each study group. In both the laparoscopic-assisted vaginal hysterectomy (LAVH) group and the Trendelenburg (TB) group urine sampling was performed at three time points: Before (PRE), during or immediately after (PER) and two hours after (POST) the procedure. Each orange vertical line represents a urine sample collection.

Urine sampling was performed for each subject in both study groups at three time points. In the LAVH group, a first urine sample was collected immediately before installing the pneumoperitoneum (PRE). A second sample was taken at deflation of the pneumoperitoneum before the closure of the abdominal ports (PER). The time interval between sample 1 and sample 2 was recorded, representing the duration of the laparoscopic procedure. The last urine sampling was performed two hours after the procedure (POST). In the Trendelenburg group, urine samples were collected before (PRE), immediately after (PER) and two hours after Trendelenburg positioning (POST) (Figure 1).

### 2.4. Urine Analyses

The urine creatinine, albumin, total protein and urea levels were measured, and the albumin/creatinine ratio (ACR) and total protein/creatinine ratio (PCR) were calculated by the clinical laboratory of ZOL for each time point. Reference values of the renal function parameters are present in Table S1.

### 2.5. Venous Doppler Ultrasonography

Two patients of the LAVH group underwent venous Doppler measurement of the hepatic veins performed by one researcher (WG) before and during the pneumoperitoneum, according to a protocol reported elsewhere [17]. According to local operation theater sterility protocol, it was not possible to perform this assessment during every procedure. However, the Hasselt University Research Team on Maternal Venous Hemodynamics already published an in depth study on the biological nature of hepatic venous Doppler flow changes [13].

### 2.6. Statistical Analysis 

Statistical analyses were performed using the Statistical Package for the Social Sciences SPSS Inc., Software version 25.0 (IBM, Chicago, Illinois, USA). The normality of distribution of the data was examined via the Shapiro-Wilk test. Data were expressed as means ± standard deviation (SD) or medians [interquartile ranges (IQR)] for parametric and non-parametric distributed data, respectively. Categorical data were expressed as the absolute number and frequency [percentage (%)]. A Pearson correlation test was used to evaluate the association between the duration of the LAVH intervention and the different renal function parameters. To compare proteinuria and albuminuria within each group before, during and after the procedure, a Wilcoxon Signed Rank or paired t-test was used. Mann-Whitney U test was performed to compare the (change in) renal function parameters between the two groups before, during and after the procedure. Categorical data were analyzed via Chi-square or Fisher’s Exact test. A significance level of alpha = 0.05 was used. 

## 3. Results

A total of 42 subjects were included in the study: Thirty-two in the LAVH group, and ten in the Trendelenburg group. Demographic and clinical characteristics of both study groups are enlisted in Table 1. The weight and medication use were higher in the LAVH group compared to the Trendelenburg group, while the latter included more nulliparous women. Detailed information with respect to pre-existing co-morbidities, parity and medication use is shown in Table S2.

The PRE, PER and POST urinary renal function parameters were compared within each study group (Table 2). When comparing PRE to PER, increases in albuminuria from 11.0 mg/L (IQR 16.8) to 77.5 mg/L (IQR 155.5) and proteinuria from 5.6 mg/dL (IQR 6.0) to 22.3 mg/dL (IQR 41.2) were measured in the LAVH group, while a decrease in proteinuria from 5.0 mg/dL (IQR 6.2) to 4.0 mg/dL (IQR 0.2), but not albuminuria, was seen in the Trendelenburg group. In the latter, urinary creatinine and urea levels significantly decreased from 60.6 mg/dL (IQR 132.4) to 22.8 mg/dL (IQR 31.2) and from 931.0 mg/dL (IQR 1953.0) to 475.0 mg/dL (IQR 858.3), respectively, which was not the case in the LAVH group. 

When comparing PRE to POST, higher levels in albuminuria [79.5 mg/L (IQR 88.8)], and proteinuria [21.0 mg/dL (IQR 28.0)] were present POST in the LAVH, but not in the Trendelenburg group. The ACR and PCR increased from PRE to PER in the LAVH group [from 18.0 mg/g creatinine (IQR 18.8) to 113.5 mg/g creatinine (IQR 136.8) and from 0.1 mg/mg creatinine (IQR 0.1) to 0.3 mg/mg creatinine (IQR 0.3), respectively], with higher ratios persisting POST-procedure [ACR = 90.5 mg/g creatinine (IQR 114.0) and PCR = 0.3 mg/mg creatinine (IQR 0.2)] compared to PRE. In the Trendelenburg group, higher levels of PCR were reported PER (0.4 ± 0.3 mg/mg creatinine), but not POST, compared to PRE (0.1 ± 0.1 mg/mg creatinine). No overall significant changes in ACR were observed in this group.

**Figure 2:**
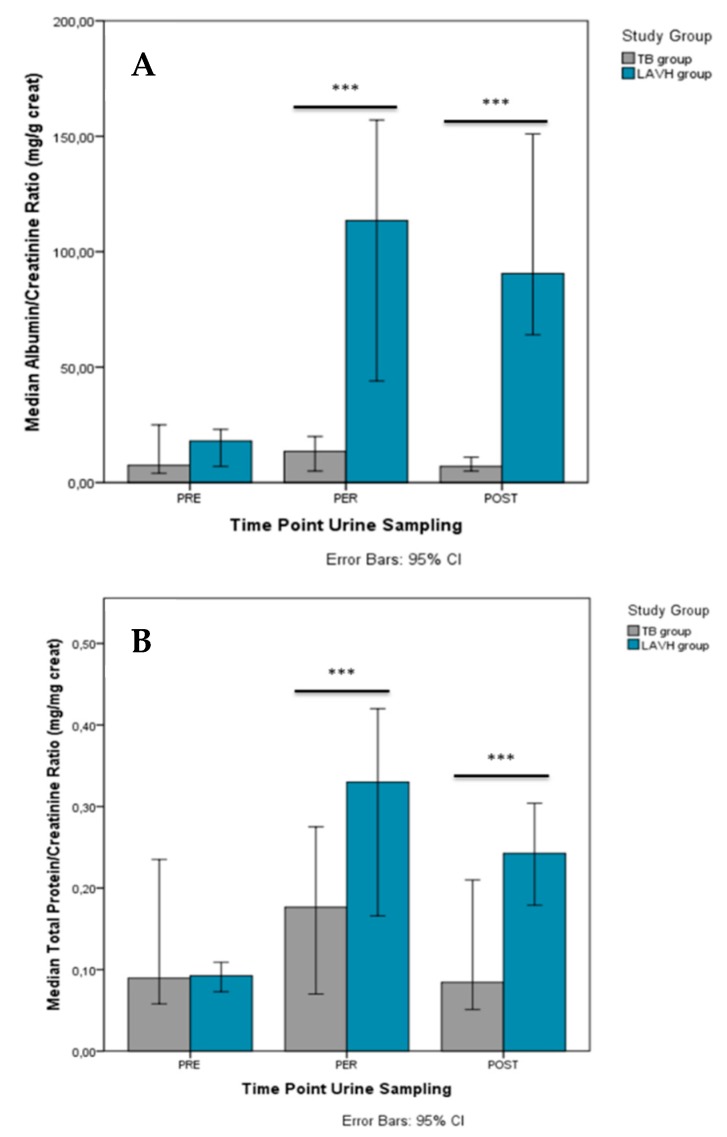
Comparison of the albumin/creatinine ratio (**A**) and total protein/creatinine ratio (**B**) between the TB and LAVH group PRE-, PER- and POST-intervention. Significant differences in the albumin/creatinine ratio and total protein/creatinine ratio between the groups were observed both PER- and POST-procedure. ***P-value < 0.001.

Comparison of the PRE, PER and POST urinary parameters between the two study groups is presented in Table S3, showing no differences before the start of the procedure. PER-procedure (i.e., LAVH or Trendelenburg intervention), urine levels of creatinine [88.8 mg/dL (IQR 88.0) vs. 22.8 mg/dL (IQR 31.2)], albuminuria [77.5 mg/L (IQR 155.50 vs. 2.0 mg/L (IQR 2.0)], proteinuria [22.3 mg/dL (IQR 41.2) vs. 4.0 mg/dL (IQR 0.2)], and urea [1216.0 mg/dL (IQR 879.0) vs. 475.0 mg/dL (IQR 858.3)] were higher in LAVH compared to controls. The differences in albuminuria and proteinuria persisted POST-procedure [79.5 mg/L (IQR 88.8) vs. 2.5 mg/L (IQR 5.5) and 21.0 mg/dL (IQR 28.0) vs. 4.0 mg/dL (IQR 1.7), respectively]. Similar results were observed with respect to the ACR (Figure 2A) and PCR (Figure 2B) between the Trendelenburg and LAVH group PER- and POST-procedure. 

The change in the level of all urinary parameters also differed significantly from PRE to PER between the two study groups (Table 3).

**Figure 3:**
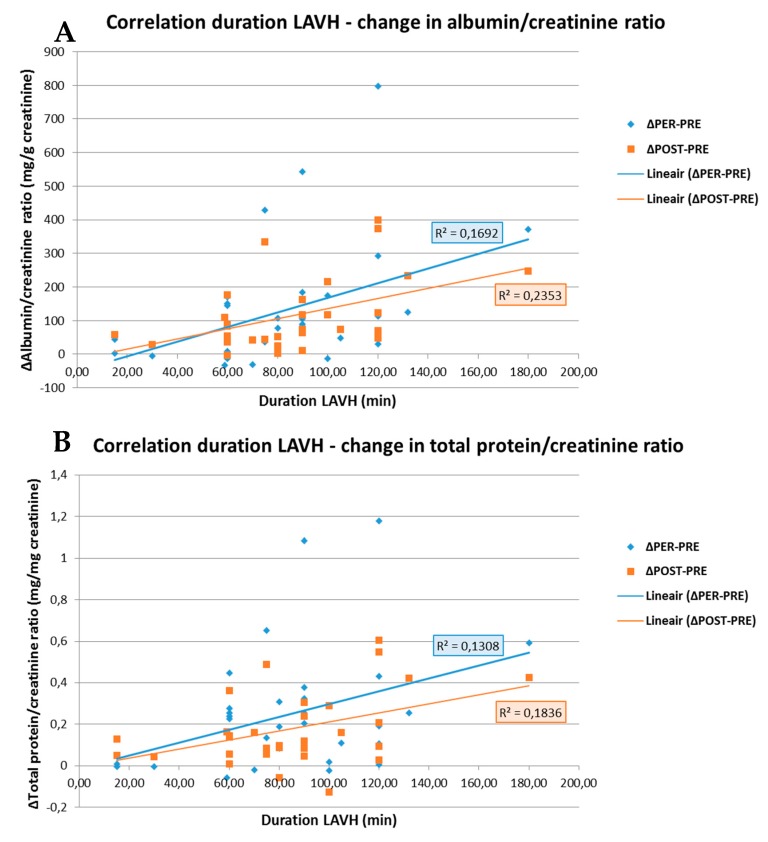
Correlation between the duration of the LAVH procedure and the change in albumin/creatinine ratio (**A**) and in total protein/creatinine ratio (**B**) PRE- to PER- and PRE- to POST-procedure. A positive correlation is observed between the duration of the LAVH procedure and the change in albumin/creatinine ratio (**A**) and total protein/creatinine ratio (**B**) from PRE- to PER-procedure, i.e., blue line (R = 0.411; p = 0.019 and R = 0.362; *p* = 0.042, respectively). The same is observed for the duration of the LAVH procedure and the change in albumin/creatinine ratio (**A**) and total protein/creatinine ratio (**B**) PRE- to POST-procedure, i.e., orange line (R = 0.485; *p* = 0.005 and R = 0.429; *p* = 0.014, respectively). *R: Pearson correlation coefficient.*

As shown in Figure 3, the change in ACR and PCR both PRE- to PER- and PRE- to POST-procedure showed a positive correlation with the duration of the surgery (R = 0.411; *p* = 0.019 and R = 0.362; *p* = 0.042 for ACR and R = 0.485; *p* = 0.005 and R = 0.429; *p* = 0.014 for PCR). No correlations between the BMI and ACR or PCR were observed in the LAVH group.

**Figure 4:**
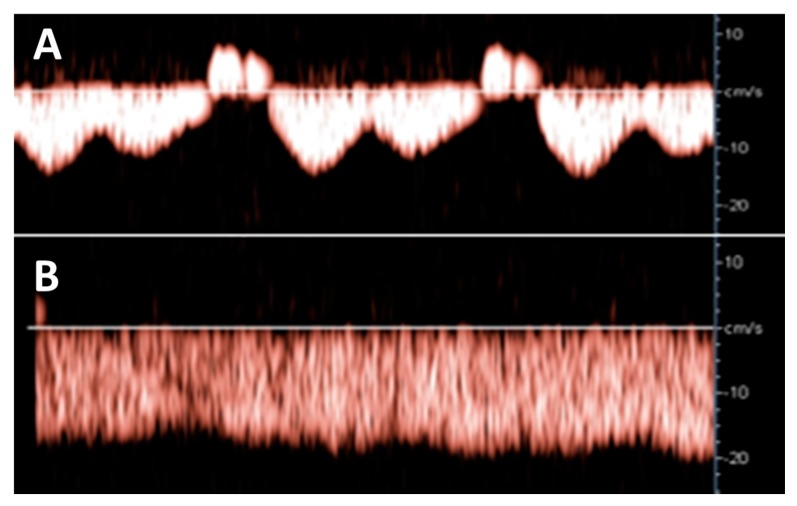
Hepatic venous Doppler measurement was performed on two patients of the LAVH group. At the start of the LAVH procedure, a triphasic pattern was observed (**A**), changing into a flat pattern following pneumoperitoneum-induced IAP during the procedure (**B**). This shift is similar to the changes observed during the Valsalva maneuver [13], and during the course of pregnancy [17].

Hepatic venous Doppler ultrasonography showed a triphasic pattern of the hepatic vein before LAVH (Figure 4A), which abruptly shifted into a flat pattern during pneumoperitoneum-induced CO_2_ insufflation at IAP values at around maximum device controlled values of 14.7 mmHg (Figure 4B).

## 4. Discussion

The main finding of this study is that a transient, iatrogenic rise in IAP during laparoscopic pneumoperitoneum (grade 1 IAH) is associated with an increase in ACR and PCR, of which the effect persisted until two hours after the procedure. This effect positively correlates with the duration of the pneumoperitoneum and co-exists with hepatic venous Doppler changes similar to those observed with the Valsalva maneuver and in uncomplicated third trimester pregnancy [13,17].

### 4.1. Increase in IAP Results in Higher ACR and PCR Through Venous Congestion

Our approach allows excluding potential interference from determinants of transient albuminuria, such as exercise, pregnancy, pyrexia, hyperglycemia, urinary infection, menstrual blood contamination. The increase of PCR observed in the Trendelenburg group can be explained by a larger decrease in urinary creatinine rather than an increase in proteinuria (Table 2), indicating the production of diluted urine. During body inversion, blood volume from the legs is shifted towards the heart by a higher venous return, inducing a higher cardiac output, and subsequently, a higher renal perfusion [18]. In LAVH, this mechanism seems to be overruled by the high IAP, causing the observed increase in ACR and PCR and excluding Trendelenburg as an etiologic explanation (Figure 2). Furthermore, with longer duration of LAVH surgery, and thus, longer increase in IAP, higher ACR and PCR were observed both PER- and POST-procedure.

In animal studies, it was demonstrated that volume expansion could reverse the IAH-associated reduction in cardiac output, although no such effect could be observed in renal output [19]. This suggested that increased renal venous pressure (RVP), rather than reduced arterial perfusion, was the main contributor to the effective renal plasma flow [20,21]. Renal arterial vasoconstriction and hypo-perfusion do occur during an increase of RVP, due to the nervous reflex response [20,22,23]. Additionally, a high RVP leads to high renal interstitial hydrostatic pressure, which is known to also relate directly to IAP. Both the arterial hypo-perfusion and the high interstitial pressure reduce the glomerular filtration rate, causing oliguria. Venous congestion also induces podocyte injury and slit diaphragm disruption, inducing albuminuria. The above mechanisms can explain the increase of albuminuria during laparoscopy, as observed in our study [24].

There is increasing evidence that tubular epithelial cells and pericytes surrounding medullary descending vasa recta play a leading role in the control of renal flow [25]. The decreased urinary flow and renal blood flow is seen during venous congestion cause tubular hypoxia and tubulointerstitial injury, which is amplified by the increase in interstitial hydrostatic pressure [11]. In congestive kidneys, pericyte detachment and extracellular matrix expansion were observed. Detached pericytes differentiate into proliferating myofibroblasts, causing interstitial fibrosis [11]. Amongst biochemical manifestations of tubulointerstitial injury is a high serum level of uric acid [25,26,27], as is also commonly seen during PE.

### 4.2. Increase in IAP During Pregnancy Might Induce Venous Congestion, Which Clinically Presents as Oliguria and Proteinuria. 

During pregnancy, IAP rises due to the growing uterus, fluid status and tissue mass. From the 2nd trimester onward, IAP values are measured within the range of IAH in non-pregnant individuals [4,12,28,29,30,31]. A pregnant woman seems to cope very well via a process of extraordinary physiologic adaptation. Pre-pregnancy BMI is positively correlated with gestational IAP [12]. Maternal hemodynamics change in pregnancy and maladaptation of the maternal cardiovascular system may result in the development of gestational hypertensive disorders. Contrary to non-proteinuric gestational hypertension, PE presents with abnormal venous Doppler measurements, more pronounced in early- than in late-onset disease, suggestive for hampered venous outflow and as such, predisposing to renal venous congestion [32].

PE is a systemic disease characterized by new onset hypertension combined with different types of organ dysfunction, usually clinically presenting as proteinuria [33]. The venous congestion and reduced arterial perfusion can lead to renal, placental and fetal ischemia [34]. In the clinical stage of PE, renal dysfunction is mostly present with reduced clearance, albuminuria and oliguria [35]. The observation that the prevalence of PE is higher in severely obese women and in multiple pregnancies, together with the increase in the frequency of PE towards the end of gestation, suggests that increased IAP is part of its pathophysiologic process [3,36,37,38,39].

Today, different phenotypes of PE have been identified according to maternal hemodynamic characteristics [40]. PE with fetal growth restriction is known as a hypertonic state with a low cardiac output and a high vascular resistance circulation, presenting predominantly before 34 weeks of gestation, and therefore, often referred to as early-onset PE (EPE) [41,42]. PE with normal fetal growth is characterized as a hypervolemia circulation with normal to high cardiac output and normal to low vascular resistance, usually presenting near term, and thus, commonly labelled as late-onset PE (LPE) [41,42,43,44,45]. Two subtypes of LPE have been reported based on a bimodal skewed distribution of birth weight [46]. Cohort studies from early pregnancy until delivery have shown that one LPE subtype presents with a cross-over from early gestational high cardiac output/low resistance circulation to late gestational low cardiac output/high resistance [47]. This cross-over phenomenon can be explained by secondary endothelial dysfunction triggered by volume (over-)load [7,48] with associated change of biochemical markers [49]. The other subtype of LPE shows a persistently high cardiac output circulation throughout gestation of which the underlying pathophysiologic mechanism is not fully understood yet [16].

**Figure 5:**
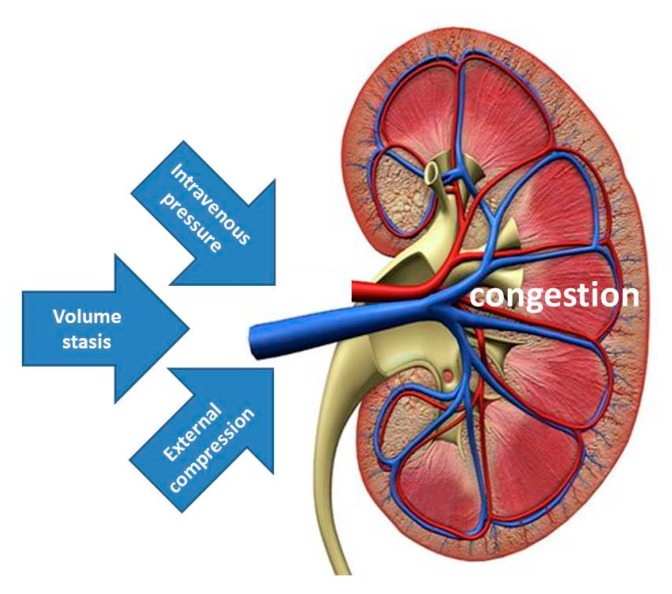
Illustration of the triad of venous congestion as a result of combinations of increased intravenous pressure, volume stasis and external compression. Increased intravenous pressure may be the predominant feature in EPE, volume stasis is the main trigger in the cross-over population reported by Bosio, whereas, external compression from increased intra-abdominal pressure is the leading cause in the persistently high cardiac output group reported by Easterling [16,47].

It has been suggested that increased IAP with external venous compression might be the mechanism explaining the spectrum of signs and symptoms of organ dysfunction and/or failure during pregnancy, labeled as PE [50]. The data from the current study support this view by illustrating that albuminuria and proteinuria may result from increased IAP. Together with the increase of renal venous pressure and stasis of intravenous volume, external venous compression completes the pathophysiologic triad of venous congestion, triggering a cascade of events, eventually leading to organ dysfunction (Figure 5). In this theoretical concept, increased intravenous pressure may be the predominant inductor of proteinuria in EPE, volume stasis is the main trigger in the cross-over population reported by Bosio, whereas, external compression from increased IAP is the leading cause in the persistently high cardiac output group described by Easterling [16,47].

### 4.3. Limitations of This Study

Our study has some limitations. We recognize the unequal and rather small sample size in both study groups. The two study groups differed in weight, parity and medication use; however, no differences in proteinuria nor albuminuria were observed PRE-procedure. IAP during LAVH was relatively low and not measured in the control group. We did not compare insufflation pressures to the golden standard, which is intravesical pressure. We also acknowledge the lack of data on other renal and cardiovascular function tests, such as creatinine clearance, cardiac output, central venous pressure, renal venous flow and renal resistivity index. However, the simultaneous change of hepatic vein Doppler pattern during insufflation of the pneumoperitoneum indicates that a rise in IAP is responsible for changes in intra-abdominal venous blood flow. As no pregnant women with or without PE were included in this study, linking our findings with the pathophysiologic process of PE is theoretical and needs confirmation from targeted clinical and experimental research. The acute rise of IAP during pneumoperitoneum does not entirely represent the slow and chronic rise of IAP during pregnancy, where multiple maternal adaptation mechanisms are active [28].

### 4.4. Future Perspectives

Our current data add to formerly reported arguments that symptoms of PE may relate to mechanisms of venous congestion. Future experimental and clinical research should focus in depth into the role of venous hemodynamics in normal and abnormal outcomes of pregnancy. Next to this, a large prospective trial is needed focusing on longitudinal observations on maternal hemodynamics from preconception, throughout pregnancy until postpartum. The results of these studies may impact current clinical management protocols of PE as different pathophysiologic background mechanisms require type-specific treatment and follow-up.

## 5. Conclusions

Pneumoperitoneum-induced IAH during laparoscopic interventions resulted in increased urinary ACR and PCR. In addition to previous pathophysiological concepts of EPE, where arterial endothelial dysfunction is the trigger, the clinical presentation of LPE seems to be mainly mediated by venous congestion.

## 6. List of Abbreviations

ACRAlbumin/creatinine ratioBMIBody-mass indexEPEEarly-onset preeclampsiaIAHIntra-abdominal hypertensionIAPIntra-abdominal pressureIQRInterquartile rangesLAVHLaparoscopic-assisted vaginal hysterectomyLPELate-onset preeclampsiaPCRProtein/creatinine ratioPEPreeclampsiaPERDuring or immediately after the procedurePREBefore the start of the procedurePOSTTwo hours after the procedureRVPRenal venous pressureTBTrendelenburgZOLZiekenhuis Oost Limburg, Genk, Belgium

## Figures and Tables

**Table 1:** Demographic and clinical characteristics of both study groups.

Characteristics	LAVH group (N = 32)	Trendelenburg group (N = 10)	*P*-value
**Age, yr**	44 (IQR 10.0)	23.5 (IQR 37.0)	NS
**Weight, kg**	75.4 ± 15.5	64.7 ± 9.8	**0.047**
**Height, m**	1.7 ± 0.1 ^a^	1.7 ± 0.1	NS
**BMI, kg/m^2^**	26.1 (IQR 6.6) ^a^	22.0 (IQR 5.7)	NS
**Smoking, n (%)** **Yes** **No**	4 (12.5) 28 (87.5)	1 (10.0) 9 (90.0)	NS
**Pre-existing co-morbidities**	10 (31.3)	1 (10.0)	NS
**Nulliparity, n (%)**	4 (12.5)	7 (70.0)	**0.003**
**Medication use, n (%)**	13 (40.6)	1 (10.0)	**0.044**

LAVH: Laparoscopic-assisted vaginal hysterectomy. N: Sample size. Yr: Years. IQR: Interquartile range. NS: Statistically non-significant difference. BMI: Body-Mass Index. n: Number of frequency. Continuous parameters are presented as median (IQR) or mean ± standard deviation depending on normality. Categorical data are presented as the number of frequency (percentage). A *P* -value <0.05 was considered a statistically significant difference and is indicated in bold. ^a^ Two missing cases. The mean ± standard deviation and median (IQR) are calculated based on 30 samples.

**Table 2:** Comparison of urinary renal function parameters PRE-, PER- and POST-procedure within each group.

Urine Test (unit)	LAVH Group (N = 32)						Trendelenburg Group (N = 10)					
	PRE	PER	POST	*P*-value †	*P*-value ‡	*P*-value §	PRE	PER	POST	*P* -value †	*P*-value ‡	*P*-value §
**URINARY CREATININE (mg/dL)**	79.8 (IQR 92.1)	88.8 (IQR 88.0)	70.3 (IQR 84.9)	NS	NS	NS	60.6 (IQR 132.4)	22.8 (IQR 31.2)	53.2 (IQR 79.5)	0.005	NS	NS
**ALBUMIN (mg/L)**	11.0 (IQR 16.8)	77.5 (IQR 155.5)	79.5 (IQR 88.8)	**< 0.001**	NS	**< 0.001**	2.5 (IQR 14.0)	2.0 (IQR 2.0)	2.5 (IQR 5.5)	NS	NS	NS
**ALBUMIN/CREATININE RATIO (mg/g creat)**	18.0 (IQR 18.8)	113.5 (IQR 136.8)	90.5 (IQR 114.0)	****< 0.001****	NS	**< 0.001**	7.5 (IQR 12.0)	13.5 (IQR 11.3)	7.0 (IQR 6.0)	NS	NS	NS
**TOTAL PROTEIN (mg/dL)**	5.6 (IQR 6.1)	22.3 (IQR 41.2)	21.0 (IQR 28.0)	**< 0.001**	NS	**< 0.001**	5.1 (IQR 6.2)	4.0 (IQR 0.2)	4.0 (IQR 1.7)	0.028	NS	NS
**TOTAL PROTEIN/CREATININE RATIO (mg/mg creat)**	0.1 (IQR 0.1)	0.3 (IQR 0.3)	0.2 (IQR 0.2)	**< 0.001**	NS	**< 0.001**	0.1 ± 0.1	0.4 ± 0.3	0.3 ± 0.2	0.018	NS	NS
**UREA (mg/dL)**	1236.0 ^a^ (IQR 1084.0)	1216.0 (IQR 879.0)	1051.5 (IQR 709.0)	NS	NS	NS	931.0 ^b^ (IQR 1953.0)	475.0 (IQR 858.3)	852.5 (IQR 908.3)	0.008	NS	NS

LAVH: Laparoscopic-assisted vaginal hysterectomy. N: Sample size. PRE: Before the procedure. PER: During or immediately after the procedure. POST: Two hours after the procedure. IQR: Interquartile range. NS: Statistically non-significant difference. Data are presented as median (IQR) or mean ± standard deviation depending on normality. A *P*-value < 0.05 was considered statistically significant and is indicated in bold. † Comparison between PRE and PER the procedure. ‡ Comparison between PER and POST the procedure. § Comparison between PRE and POST the procedure. ^a^ One missing case; the median (IQR) is calculated based on 31 samples. ^b^ One missing case; the median (IQR) is calculated based on nine samples.

**Table 3:** Change in renal function parameters over time for both the intervention and the control group.

Urine Test (unit)	PER-PRE			POST-PER			POST-PRE		
	LAVH Group (N = 32)	TB Group (N = 10)	*P*-value †	LAVH Group (N = 32)	TB Group (N = 10)	*P*-value ‡	LAVH Group (N = 32)	TB Group (N = 10)	*P*-value §
**∆CREATININE (mg/dL)**	6.6 (IQR 52.8)	−29.7 (IQR 86.3)	0.002	−8.6 (IQR 78.4)	21.1 (IQR 66.7)	0.026	−5.8 ± 75.0	−6.9 ± 95.4	NS
**∆ALBUMIN (mg/L)**	63.0 (IQR 148.5)	−0.5 (IQR 12.5)	**< 0.001**	−2.5 (IQR 138.5)	0.0 (IQR 5.5)	NS	63.0 (IQR 92.5)	−0.5 (IQR 7.8)	**< 0.001**
**∆ALBUMIN/CREATININE RATIO (mg/g creat)**	85.0 (IQR 151.3)	1.5 (IQR 3.5)	**< 0.001**	1.0 (IQR 96.5)	−3.0 (IQR 12.3)	NS	72.0 (IQR 109.0)	−1.0 (IQR 11.0)	**< 0.001**
**∆TOTAL PROTEIN (mg/dL)**	15.7 (IQR 36.1)	−1.1 (IQR 6.2)	**< 0.001**	−0.5 (IQR 31.0)	0.0 (IQR 1.5)	NS	12.5 (IQR 23.0)	−1.1 (IQR 4.8)	**< 0.001**
**∆TOTAL PROTEIN/CREATININE RATIO (mg/mg creat)**	0.2 (IQR 0.3)	0.1 (IQR 0.1)	**0.033**	−0.1 (IQR 0.3)	−0.1 (IQR 0.2)	NS	0.1 (IQR 0.2)	−0.0 (IQR 0.1)	**< 0.001**
**∆UREA (mg/dL)**	−46.5 (IQR 376.8)	−279.0 (IQR 654.8)	NS	−17.5 (IQR 605.5)	199.5 (IQR 917.8)	NS	−225.0 ± 773.0	−168.1 ± 1114.4	NS

PRE: Before the procedure. PER: During or immediately after the procedure. POST: Two hours after the procedure. LAVH: Laparoscopic-assisted vaginal hysterectomy. TB: Trendelenburg. N: Sample size. ∆: Change. IQR: Interquartile range. NS: Statistically non-significant difference. Data are presented as median difference (IQR). A *P*-value < 0.05 was considered statistically significant and is indicated in bold. † Comparison of the change in urine sampling results from PRE to PER the procedure between the groups. ‡ Comparison of the change in urine sampling results from PER to POST the procedure between the groups. § Comparison of the change in urine sampling results from PRE to POST the procedure between the groups.

## Supplementary Materials

The following are available online at https://www.mdpi.com/2077-0383/9/2/487/s1, Table S1: Reference ranges of the analyzed urinary renal function parameters. Table S2: Parity and detailed medication usage of subjects in both study groups. Table S3: Urine analysis of both the intervention and the control group at different time points.
